# Label-Free, Impedance-Based Biosensor for Kidney Disease Biomarker Uromodulin

**DOI:** 10.3390/s23249696

**Published:** 2023-12-08

**Authors:** Kunj Vora, Norbert Kordas, Karsten Seidl

**Affiliations:** 1 Fraunhofer Institute for Microelectronic Circuits and Systems, 47057 Duisburg, Germany; norbert.kordas@ims.fraunhofer.de (N.K.); karsten.seidl@ims.fraunhofer.de (K.S.); 2 Department of Electronic Components and Circuits, University of Duisburg-Essen, Forsthausweg 2, 47057 Duisburg, Germany

**Keywords:** impedance spectroscopy, atomic layer deposition, tantalum pentoxide, uromodulin, chronic kidney disease

## Abstract

We demonstrate the development of a label-free, impedance-based biosensor by using a passivation layer of 50-nm tantalum pentoxide (Ta_2_O_5_) on interdigitated electrodes (IDE). This layer was fabricated by atomic layer deposition (ALD) and has a high dielectric constant (high-κ), which improves the capacitive property of the IDE. We validate the biosensor’s performance by measuring uromodulin, a urine biomarker for kidney tubular damage, from artificial urine samples. The passivation layer is functionalized with uromodulin antibodies for selective binding. The passivated IDE enables the non-faradaic impedance measurement of uromodulin concentrations with a measurement range from 0.5 ng/mL to 8 ng/mL and with a relative change in impedance of 15 % per ng/mL at a frequency of 150 Hz (log scale). This work presents a concept for point-of-care biosensing applications for disease biomarkers.

## 1. Introduction

Point-of-care biosensors present a promising solution for the regular monitoring of biomarkers for the early diagnosis of diseases like cancer [1], Alzheimer’s disease [2] and metabolic disorders [3]. Biosensors employ either a labeled approach or a label-free approach as the underlying principle. Comparing both approaches, the label-free approach requires fewer sample preparation steps [4].

Electrical impedance spectroscopy (EIS) offers a label-free biosensing principle with a wide detection range and sensitivity [5,6,7,8]. EIS is classified into the faradaic and non-faradaic methods. The faradaic method measures the charge transfer resistance and the double-layer capacitance at the electrode–electrolyte interface over a frequency range. This method requires redox couples to observe the changes, which makes it unsuitable for point-of-care measurements [9]. The non-faradaic (capacitive) method involves the passivation of the electrode, which eliminates the need for a redox couple to generate a signal, and measures the change in capacitance [10,11,12].

Non-faradaic sensors require a thin, pin-hole free passivation layer to obtain maximum capacitance [9]. The biomarkers bind to the functionalized passivation layer. The total capacitance ($C_{t}$) is the series connection of the passivation layer capacitance ($C_{p}$), the recognition layer capacitance ($C_{r}$) and the diffuse layer capacitance ($C_{d}$), shown in Equation (1) [9,13].

(1) $$\frac{1}{C_{t}}=\frac{1}{C_{p}}+\frac{1}{C_{r}}+\frac{1}{C_{d}}$$

Regular monitoring of kidney biomarkers could help in the early diagnosis of kidney disease in patients with hypertension and diabetes [14,15]. The glomerular filtration rate (GFR), albuminuria and cystatin C are the gold standards for kidney disease diagnosis. However, diagnosis through these methods entails an indirect and invasive approach [16].

A urine biomarker enables easier, non-invasive and regular monitoring of the kidneys. Uromodulin, produced in the ascending limb of Henle’s loop of the nephron, is the most abundant protein in urine (30–180 µg/mL) [17]. Several studies have found uromodulin to be an important biomarker for kidney tubular damage and to predict progressive kidney disease in addition to the established biomarkers [18,19,20,21,22]. The enzyme-linked immunosorbent assay (ELISA) measures uromodulin concentrations from urine or blood quantitatively. However, the lengthy laboratory procedure mandates skilled personnel to obtain accurate results. Previous studies describe biosensor concepts for other kidney biomarkers but not for uromodulin [23,24].

**Figure 1:**
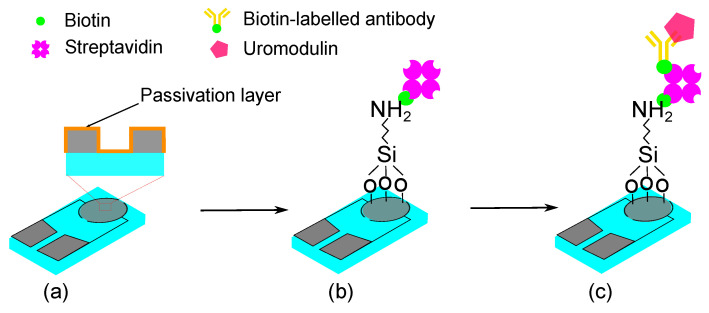
Schematic representation of the sensor concept for uromodulin detection with passivated functionalized IDE: (**a**) passivated IDE, (**b**) silanization and biotin–streptavidin immobilization, (**c**) biotin-labelled antibody immobilization and uromodulin immobilization.

Our aims were to develop a label-free, impedance-based, single-use biosensor by depositing a passivation layer on interdigitated electrodes (IDE), through the atomic layer deposition (ALD) of tantalum pentoxide (Ta_2_O_5_) (Figure 1). We immobilized uromodulin on the sensor from synthetic urine samples and measured the impedance over frequency.

## 2. Methods

**Figure 2:**
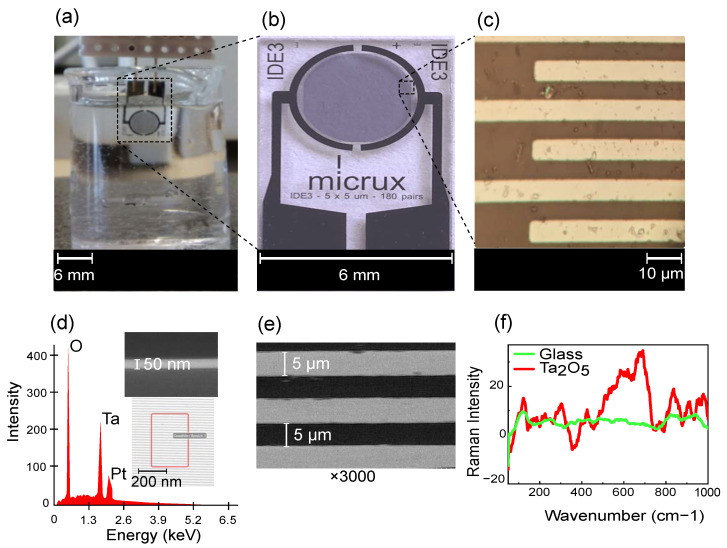
(**a**) Measurement setup with IDE dipped in PBS solution, (**b**) enlarged image of sensor element from Micrux Technologies with (**c**) microscopic image of the IDE fingers (*w* = 5 µm, *g* = 5 µm), (**d**) EDS characterization showing composition of IDE with EDS scanning area and SEM image (cross-section) of 50-nm Ta_2_O_5_ on substrate, (**e**) SEM characterization of IDE (top-view) with ×3000 magnification and (**f**) Raman spectra of the IDE substrate (glass) before and after passivation with Ta_2_O_5_ using excitation wavelength of 532 nm (baseline-corrected and smoothed spectrum).

The sensor element is a thin-film platinum IDE (ED-IDE3-Pt) purchased from Micrux Technologies (Asturias, Spain). The IDE has a finger length (*l*) of 2800 µm, finger width (*w*) and finger gap (*g*) of 5 µm (Figure 2b,c). We passivated these IDE with 50-nm Ta_2_O_5_ using atomic layer deposition. Considering the IDE geometry, the electric fields primarily concentrate below 10 µm (w+g) [25]. Therefore, a passivation thickness of 50 nm proves to be suitable for a capacitive sensor.

### 2.1. Atomic Layer Deposition (ALD)

ALD entails sequential layer-by-layer material deposition, resulting in a nanometer-scale thin film [26]. ALD produces uniform, conformal, thin films in comparison to other physical and chemical deposition techniques [27]. The deposition requires two precursors, which interact with the substrate surface sequentially in the vapor phase under vacuum. Tert-butlimido tris (Dimethylamido) tantalum (TBTMET) is used as the first precursor and water is the second precursor. The deposition temperature is 120 °C for 560 cycles, ensuring the required layer thickness, and the thickness is confirmed using scanning electron microscopic (SEM) images (Figure 2d (inset)).

The ALD technique for Ta_2_O_5_ deposition offers the advantage of pin-hole free layers, with a high dielectric constant (high-κ) of 18–25 [28,29]. The presence of Ta_2_O_5_ is confirmed with EDS spectra characterization (Figure 2d), showing the oxygen at weight and atomic percentages 31.1/68.4%, tantalum 32.8/6.4% and platinum 24.1/4.3% as prominent peaks. Small quantities of other elements, like carbon (4.4/12.8%) and silicon (6.3/7.9%), are also seen. SEM characterizes the dimensions of the IDE fingers (Figure 2e) and the thickness of the passivation layer on the sensor. The sensor is also characterized with Raman spectroscopy, showing a clear change before and after passivation (Figure 2f). The spectrum is comparable to that of amorphous Ta_2_O_5_ [30].

### 2.2. Sensor Functionalization

Sensor functionalization creates functional groups on the sensor surface for the selective binding of the biomarker. The functionalization protocol for Ta_2_O_5_ has been explained by [31,32]. We purchased the reagents 3-aminopropyl-trimethoxysilane, toluene, biotin, streptavidin, Sigmatrix synthetic urine and phosphate-buffered saline (PBS) from Sigma-Aldrich (St louis, MO, USA). The ELISA kit for uromodulin, from BioVendor (Laboratorni medicina a.s., Brno, Czech Republic), consisted the biotin-labelled polyclonal human uromodulin antibodies and polyclonal human uromodulin, which we used to immobilize uromodulin on the sensor surface.

The passivated IDE were first washed with deionized water followed by isopropyl alcohol and placed in the oven at 110 °C to remove moisture. We treated the IDE with oxygen plasma to remove any organic impurities and make the surface hydrophilic. A 2% *v*/*v* silane solution was prepared with 3-aminopropyltrimethoxysilane in anhydrous toluene, and the IDE were immersed in this solution for 1 h heated at 100 °C. We washed the substrates with toluene to remove the excess unbound silane and placed the IDE in the oven to dry at 100 °C for another 1 h. The substrates were washed with deionized water and dried under an air stream.

The IDE were treated with a biotin solution in PBS (5 mg/mL) for 1 h, followed by a streptavidin solution prepared in PBS (1 mg/mL) for 1 h. The biotin–streptavidin protocol was adapted from [33]. This layer forms the base to bind the biotin-labelled uromodulin antibodies on the IDE surface. The antibodies (10×) were diluted in the dilution buffer provided in the ELISA kit. Diluted antibodies (100 µL) were allowed to react with the sensor surface for 1 h. After each step, the IDE were rinsed with deionized water to remove the unbound reagents. The different uromodulin concentrations from the ELISA kit were prepared in synthetic urine and drop-cast (50 µL) on the IDE surface for 1 h.

We measured the impedance of the IDE after each functionalization step by dipping it in 1× PBS solution (Figure 2a). The Keysight impedance analyzer E4990a (Santa Rosa, CA, USA) was used for all the measurements. The IDE, after dipping in PBS, gave a stable impedance spectrum after 2 min. The impedance analyzer was set at an excitation voltage of 10 mV and for a frequency range between 20 Hz and 1 MHz. All measurements were performed at room temperature.

## 3. Results and Discussion

**Figure 3:**
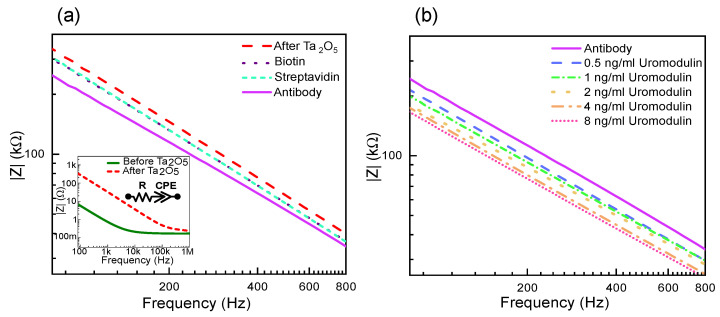
Measurement results showing absolute impedance for layer formations on IDE: (**a**) before and after Ta_2_O_5_ layer with the equivalent circuit (inset) followed by biotin, streptavidin and antibody layers, and (**b**) uromodulin concentrations in artificial urine.

**Figure 4:**
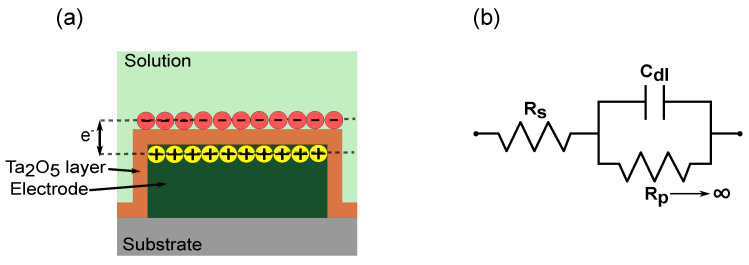
Non-faradaic principle represented by (**a**) schematic of electrode passivated with Ta_2_O_5_ where transfer of charges with solution is prevented and charges are accumulated at the interface instead, and (**b**) equivalent circuit representation of the electrochemical mechanisms, with series resistor ($R_{s}$) double-layer capacitor (*C*_dl_) and polarization/charge transfer resistance ($R_{p}$) tending to infinity.

The Ta_2_O_5_ passivated electrode shows capacitive behavior as compared to before passivation, which is due to the permittivity and thickness of the Ta_2_O_5_ layer (Figure 3a (inset)). The impedance spectroscopy measurement results are fitted to an equivalent circuit with a series resistor ($R_{s}$), which is the sum of the solution resistance and polarization resistance, the interface capacitance effect as a double-layer capacitor (*C*_dl_) and the polarization/charge transfer resistance ($R_{p}$) at the electrode–electrolyte interface in parallel to *C*_dl_. The passivated electrode prevents the transfer of charges at the electrode–electrolyte interface (Figure 4).

This leads to $R_{p}$ tending to infinity, and the impedance of the sensor is dependent on the $R_{s}$ and *C*_dl_. Therefore, for non-faradaic measurements, the equivalent circuit is a series connection of $R_{s}$ and *C*_dl_. The advantage is that the impedance is solely dependent on the protein immobilized, without requiring additional redox couples to enhance the impedance change, further supporting the label-free approach. If the measurement solution is kept constant, the impedance change depends on the changing *C*_dl_ values.

In cases where the capacitive behavior is not ideal, it is represented by a frequency-dependent element called a constant phase element (CPE), instead of *C*_dl_ for the double layer, which is defined by a value and the coefficient of CPE. The impedance CPE is defined as in (2).

(2) $$Z_{\mathrm{CPE}}=\frac{1}{{\left(j\pi f\right)}^{\alpha }Q}$$

Here, *j* is the imaginary part, *f* is the frequency, *Q* is the CPE value and α is the coefficient of CPE.

The α has values between 1 and 0, where 1 indicates ideal capacitive behavior and 0 indicates an ideal resistor. The CPE behavior is due to the accumulation of charge species on the surface and is usually found in electrochemical double-layer capacitors. The measurement results in this work are represented as a resistor and CPE in series. The impedance of the sensor is defined as in (3).

(3) $$Z=R+\frac{1}{{\left(j\pi f\right)}^{\alpha }Q}$$

The α observed by fitting the data to the equivalent circuit is 0.8 before the tantalum pentoxide layer and 0.9 after the tantalum pentoxide layer. The deposition of tantalum pentoxide increases the range of capacitive behavior of the sensor by eliminating the influence of the charge transfer resistance.

Figure 3a also shows the effect on impedance due to the different layer formations on the sensor surface. The oxide layer accumulates charges at the electrode–electrolyte interface, forming a double layer [34]. The impedance decreases with each layer formed as the area for charge accumulation increases due to more molecules bound to the sensor surface. Similarly, the IDE sensor shows a decrease in impedance with an increase in the concentration of uromodulin immobilized from artificial urine samples (Figure 3b). The protein has varying permittivity values along its structure, with a compact hydrophobic core [35]. The core has the lowest permittivity and, moving towards the periphery, the proteins interact with water to form hydration shells, which have permittivity values close to that of water [36]. The protein–antibody binding alters the double-layer charge distribution and displacement of water molecules. This causes negative charge accumulation at the interface, leading to an impedance decrease [37,38].

**Figure 5:**
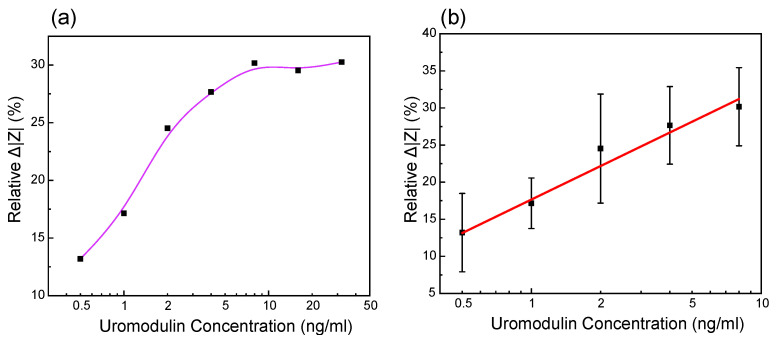
Measurement results of the relative change in impedance values of uromodulin in artificial urine at the frequency of 150 Hz of different IDE against different uromodulin concentrations in (**a**) dynamic range 0.5 ng/mL–32 ng/mL and (**b**) linear fitting of values in range 0.5 ng/mL–8 ng/mL (n = 3, relative Δ|Z|(%) = 17.6+15 log [uromodulin concentration], R^2^ = 0.976).

Three different IDE were used to measure the uromodulin concentrations prepared in artificial urine. The impedance spectrum stabilized after 2 min, which we considered as the response time of the sensor. We measured the uromodulin concentrations in the range of 0.5 ng/mL–32 ng/mL and calculated the relative change in impedance magnitude using (4) at 150 Hz (Figure 5a).

(4) $$\mathrm{Relative}\Delta |Z|\left(\%\right)=\frac{{\left|Z\right|}_{\mathrm{conc}.}-{\left|Z\right|}_{\mathrm{blank}}}{{\left|Z\right|}_{\mathrm{blank}}}$$

Here, ${\left|Z\right|}_{\mathrm{conc}.}$ is the magnitude of impedance for the uromodulin concentrations and ${\left|Z\right|}_{\mathrm{blank}}$ is the magnitude of impedance for only the antibody on the sensor surface (before uromodulin). The sensor shows a linear increase in the Δ|Z|(%) in the range of 0.5 ng/mL–32 ng/mL of uromodulin (Figure 5b). By employing this approach, our biosensor demonstrates a relative Δ|Z|(%) of 15 per ng/mL of uromodulin (log scale). The average coefficient of variation (CV %) calculated from the mean and standard deviation values over the inter-sensor results was found to be 10.7 %.

The error in the calibration curve is mainly due to the noise in the measurement fluid. We performed all the measurements by submerging the IDE in a beaker with PBS, which would contain microbubbles due to dissolved gasses when in contact with the environment. Impedance measurements are highly sensitive to the air bubbles that are formed at the electrode–electrolyte interface [13]. In the future, we aim to reduce this noise by introducing a microfluidic channel over the sensing region to limit the volume of the measurement fluid in contact with the sensing region. The fluid can also be filtered and degassed before it is placed in contact with the sensor.

**Figure 6:**
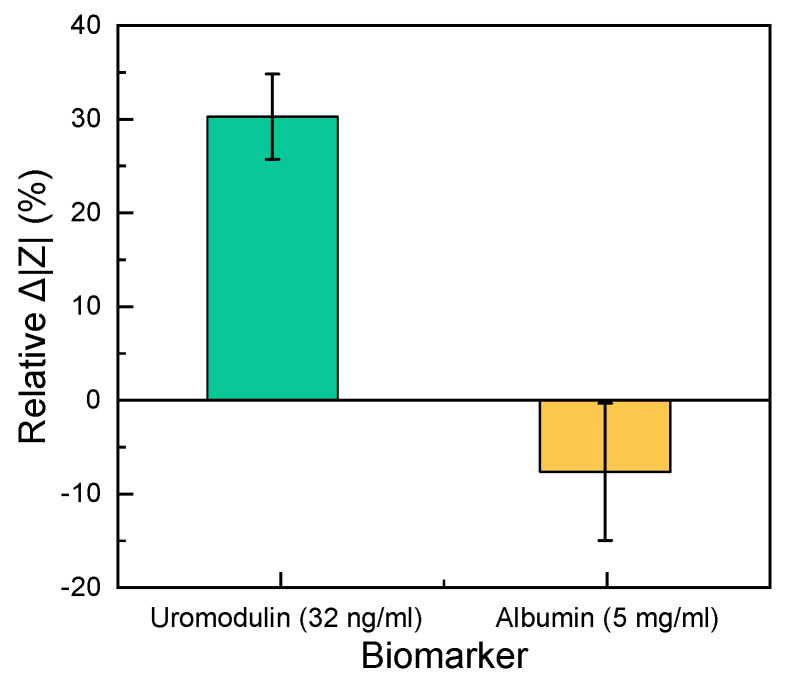
Comparison of the relative change in impedance between 32 ng/mL uromodulin and 5 mg/mL albumin in artificial urine solution (n = 3). The zero line shows the blank measurement.

Under the normal functioning of the kidneys, a high concentration of uromodulin is present in the urine (30–180 µg/mL). When the kidney function is reduced, the uromodulin concentration in the urine decreases. This situation is the opposite to that observed in the case of albumin. Albumin is a conventional biomarker for kidney damage, which could be a source of interference for the proposed uromodulin sensor. Under the normal functioning of the kidneys, albumin should not be present in the urine (<30 µg/mL). We observed the influence of albumin on the impedance by employing a clinically relevant albumin concentration (5 mg/mL) and compared the response with a uromodulin control sample (32 ng/mL) on different sensors. The relative change in impedance for uromodulin was 30 times higher than that of albumin from a blank measurement at 150 Hz (Figure 6). For the given point-of-care application, this difference in impedance change would be sufficient to distinguish between normal and abnormal kidney function.

## 4. Conclusions

We investigated a 50-nm insulating layer of tantalum pentoxide using the ALD technique on an IDE for label-free and non-faradaic biosensing. Our biosensor demonstrated a relative Δ|Z|(%) of 15 per ng/mL of uromodulin concentration and exhibited a linear range from 0.5 ng/mL to 8 ng/mL in artificial urine, operating at a frequency of 150 Hz (log scale). Non-faradaic electrochemical sensors require a thin, pin-hole-free, high-κ passivation layer [9]. The 50-nm tantalum pentoxide layer achieved in this work is 40 times thinner than what is achievable by magnetron discharge sputtering [39].

Similar dynamic ranges are observed in other non-faradaic sensors [10]. The minimally detectable concentration for our sensor is 0.5 ng/mL, which is similar to that of the commercial ELISA test [22,40]. In comparison to the ELISA test, our sensor works without additional labels. It also provides results three times faster than the commercial ELISA test.

The sensor’s performance was tested in artificial urine solutions (containing salts found in urine, urea and creatinine), to be as close to reality as possible. We tested the sensor’s selectivity by comparing the impedance change with albumin to determine its performance against unspecific binding. A uromodulin concentration of 32 ng/mL showed a 30% relative change in impedance from the blank as compared to albumin. Even the lowest concentration of uromodulin measured (0.5 ng/mL) showed a 13% relative change in impedance (Figure 5b), which was higher than that of the concentrated albumin.

The development of a uromodulin biosensor could have potential application value in clinical diagnostics, the monitoring of kidney function and the study of renal diseases. It could provide a non-invasive and a point-of-care method of detecting and quantifying the uromodulin levels in urine, offering valuable insights into kidney health and disease progression.

This work offers the opportunity to expand the application capabilities for the single-use sensor with measurements with other biomarkers. As next steps, we will test the selectivity of the sensor to other biomarkers present in urine. Additionally, we will investigate the ALD technique with other oxides on various sensor geometries and substrate materials, to assess and compare their performance. The label-free sensing approach could be a promising tool for the rapid diagnosis of other chronic illnesses or even cancer.

## Data Availability

Experimental data are available upon request.
